# Liver microwave ablation: a systematic review of various FDA-approved systems

**DOI:** 10.1007/s00330-018-5842-z

**Published:** 2018-11-30

**Authors:** Simeon J. S. Ruiter, Wouter J. Heerink, Koert P. de Jong

**Affiliations:** 1 Department of HPB Surgery and Liver Transplantation, University of Groningen, University Medical Center Groningen, Groningen, Netherlands; 2 Department of Radiology, University of Groningen, University Medical Center Groningen, Groningen, Netherlands; 3 Center for Medical Imaging, University of Groningen, Groningen, Netherlands

**Keywords:** Microwaves, Ablation techniques, Electromagnetic radiation, Tumor volume, Liver diseases

## Abstract

**Objectives:**

The aim of the present study is to analyze preclinical and clinical data on the performance of the currently US Food and Drug Administration (FDA)–approved microwave ablation (MWA) systems.

**Methods:**

A review of the literature, published between January 1, 2005, and December 31, 2016, on seven FDA-approved MWA systems, was conducted. Ratio of ablation zone volume to applied energy R(AZ:E) and sphericity indices were calculated for ex vivo and in vivo experiments.

**Results:**

Thirty-four studies with ex vivo, in vivo, and clinical data were summarized. In total, 14 studies reporting data on ablation zone volume and applied energy were included for comparison R(AZ:E). A significant correlation between volume and energy was found for the ex vivo experiments (*r* = 0.85, *p* < 0.001) in contrast to the in vivo experiments (*r* = 0.54, *p* = 0.27).

**Conclusion:**

Manufacturers’ algorithms on microwave ablation zone sizes are based on preclinical animal experiments with normal liver parenchyma. Clinical data reporting on ablation zone volume in relation to applied energy and sphericity index during MWA are scarce and require more adequate reporting of MWA data.

**Key Points:**

*• Clinical data reporting on the ablation zone volume in relation to applied energy during microwave ablation are scarce.*

*• Manufacturers’ algorithms on microwave ablation zone sizes are based on preclinical animal experiments with normal liver parenchyma.*

*• Preclinical data do not predict actual clinical ablation zone volumes in patients with liver tumors.*

## Introduction

Thermal ablation such as microwave ablation (MWA) is widely applied for the treatment of liver tumors. Thermal ablation alone or in combination with partial hepatectomy increases the number of intentionally curative treatments in patients in whom partial hepatectomy alone is not an option because of anatomical or functional reasons. Especially in patients with recurrent colorectal liver metastases (CRLM) after previous partial hepatectomy, thermal ablation increases the number of patients who could benefit from repeated procedures [1, 2]. However, the major problem of thermal ablation is incomplete ablation leading to ablation site recurrences (ASR), for which a clear definition should be used [3]. ASR is shown in 60% of patients with a lesion > 5 cm compared to 26% for 3–5 cm lesions and 16% for lesions < 3 cm [4].

Shape and volume of the ablation zone after MWA are depending on physical parameters as thermal conductivity, perfusion rate of the liver parenchyma. These parameters can be different in human liver tissue due to fibrosis, cirrhosis or steatosis [5, 6]. Planning for ablation is partially based on manufacturer-initiated working algorithms in combination with personal experience of the operator. These algorithms, which try to predict the three-dimensional diameter of the ablation zone in relation to the amount of applied energy, are often based on experiments which have serious shortcomings preventing a reliable translation to daily clinical practice. These shortcomings are the result of studies performed in (a) porcine or bovine liver (as opposed to human liver), (b) liver parenchyma (as opposed to tumors), (c) normal liver parenchyma (as opposed to cirrhotic, steatosis, or otherwise non-normal liver parenchyma in humans), and (d) non-perfused ex vivo livers (as opposed to perfused in vivo human livers with variable arterial and portal blood flow). These differences affect the way in which the applied energy is transferred into heat, resulting in highly unpredictable ablation zone volumes [6]. Additionally, despite several individual papers reporting on these shortcomings, a systematic review on this topic is lacking. The aim of this review is to analyze preclinical and clinical data on the performance of the US Food and Drug Administration (FDA)–approved MWA systems. [7]

## Methods

### Literature search and collected data

A systematic review of seven FDA-approved microwave ablation systems was performed in accordance with the PRISMA statement (Table 1) [13]. Literature published between 1 January 2005 and 1 January 2017, was searched  on Scopus including MEDLINE and EMBASE database, using the keywords “microwave ablation” AND “liver.” Retrieved studies were assessed for eligibility based on title and abstract—full papers were obtained and assessed in detail. Studies were included if (a) data on diameter or volume of the ablation zone—based on imaging or pathology—was reported, (b) ablation procedures were performed with FDA-approved MWA systems (Table 1), and (c) the amount of applied energy was reported and (d) were published in English. A data extraction form was used to collect relevant information including type of study (ex vivo, in vivo, or clinical), subject (porcine, bovine, sheep, or human), malignancy (none, primary, or secondary), device, parameters/outcomes, and measurement of ablation zone dimensions (on imaging or gross pathology). Data collection and analysis of unequivocal literature was performed by one researcher. Equivocal papers or data were discussed with co-authors until consensus was obtained. The included studies were categorized in preclinical (animal) and clinical (patient) studies. Preclinical studies were subdivided in two subcategories: ex vivo and in vivo studies (Table 1).

**Table 1:** Number of published preclinical and clinical studies using various MWA devices

	AngioDynamics, Acculis MTA	HS Medical, Amica™	Ethicon, NeuWave	MedWaves, AveCure™	Medtronic, Evident™	Medtronic, Emprint™	Perseon, MicroThermX™	Total
	A	B	C	D	E	F	G	
US FDA clearance	January 2006	September 2009	October 2010	January 2008	December 2008	April 2014	August 2010	
frequency (MHz)	2450	2450	2450	915	915	2450	915	
Ex vivo (perfused)	3 (0)	8 (0)	5 (1)	3 (1)	3 (1)	0 (0)	1 (1)	23 (4)
In vivo	4	3	3	0	1	0	0	11
Clinical studies	0	4	1	1	0	3	0	9
Total	7	15	9	4	4	3	1	43^a^

^a^The number of total studies is higher than the number of included published papers. Three papers published ex vivo and in vivo data [8–10]. One paper published ex vivo, in vivo, and clinical data [11]. One paper published ex vivo data of four MWA devices [12]. These are counted as individual studies

### Ablation zone volume and applied energy

Ablation zone volume as reported in the selected papers was recorded. If only long-axis diameter (*LAD*) and short-axis diameters (*SAD*) were recorded, the ablation zone volume was estimated, assuming ellipsoid morphology by $V=\frac{4}{3}\pi \left( LAD/2\right){\left( SAD/2\right)}^2$. Papers with only one diameter of the ablation zone were excluded for quantitative comparison. Sphericity index of ablation zones with reported SAD and LAD was calculated by *SI* = *SAD*^2^/*LAD*^2^.

The cumulative applied energy was determined by multiplying the power level (Watt), as set on the MWA generator, and the ablation time (seconds). The relation between applied energy and ablation zone volume is the only way to quantitatively compare the various published reports. To this end, the ratio of ablation zone volume to applied energy R(AZ:E) of each ablation experiment was calculated by dividing the ablation zone volume (mL) by the applied energy (kJ). The correlation coefficient was determined using IBM SPSS Statistics version 23 (IBM Corporation). The mean volume and applied energy of the subgroups were represented in a bubble chart. The sizes of the bubbles are determined by the sample size of the subgroup.

## Results

**Fig. 1:**
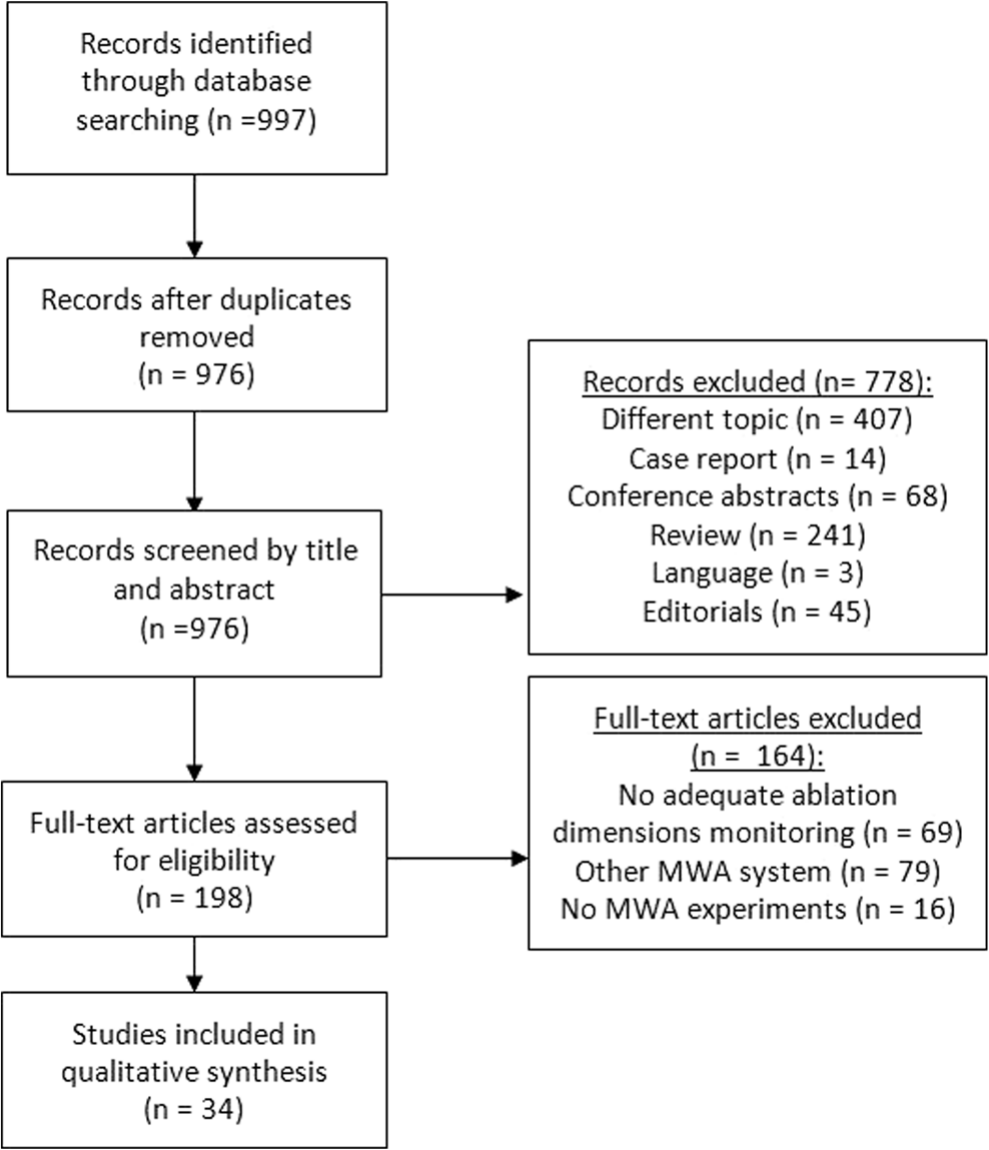
Flow chart of article selection process

Figure 1 shows a flow diagram of study identification and the exclusion process, resulting in 34 eligible studies [8–12, 14–42] (Tables 2, 3, and 4). Cross-referencing of the identified studies did not reveal any additional papers. In three studies, both ex vivo and in vivo data were published [8–10], and these were counted as six separate studies in Table 1. In one study, ex vivo, in vivo, and clinical data were presented [11], and these were counted as three separate studies. Also, in one paper, the ex vivo data of four MWA devices were presented, and these were counted as four separate studies [12]. Assessment of publication bias (overreporting of significant positive results) is not applicable for this review, because the data is not presented as negative or positive. We performed a partial correction for the heterogeneity (variability in the study characteristics) in the analyzed studies by reporting stratified results (for instance ex vivo vs. in vivo and animal vs. human).

**Table 2:** Included ex vivo studies for qualitative analysis

Author + year	Subjects	MWA system	Ablation zone measured by	Ablation protocol (energy and time)	Ablation diameter (cm)	Ablation volume (mL)	Sphericity index
Hines-Peralta et al 2006 [9]	Bovine 120 ablations	A	GP	50–150 W 4–20 min	3.0 × 3.5–7.6 × 12.3	16.5–372.0^a^	0.33–0.75
Lopresto et al 2012 [35]	3 bovine liver	B	GP	30 W 10 min	3.7 ± 0.4 × 3.2 ± 0.4	19.8^a^	0.75
Sommer et al 2012 [39]	8 porcine 15 ablations	B	GP	20–105 W 5 min	2.3 × 4.0–3.5 × 6.7	11.1–42.3	0.27–0.33
Lubner et al 2012 [10]	Bovine 18 ablations	C	GP	135 W 4–16 min	3.5 ± 0.2–4.8 ± 0.2	–	–
Hoffmann et al 2013 [12]	13 bovine 108 ablations	ABDE	GP	Manufacturer recommendations	A: 4.34 B: 4.55 D: 4.09 E: 2.68	A: 57.5 B: 72.3 D: 56.0 E: 17.1	A: 0.75 B: 0.68 D: 0.58 E: 0.64
Collettini et al 2013 [41]	50 cuboid bovine	E	MR	45 W 7 min	–	7.3 ± 2.1 (seq1) 4.7 ± 1.6 (seq2)	–
Dodd et al 2013 [37]	15 blood-perfused bovine livers 60 ablations	G	GP	60 W 10 min	4.73 ± 0.21 × 2.93 ± 0.10–5.22 ± 0.17 × 2.82 ± 0.12	21.30 ± 0.95–22.6 ± 1.53	0.29–0.38
Liu et al 2014 [17]	6 bovine	C	GP	100 W 10 min	6.45 ± 0.36 × 3.88 ± 0.2	50.8^a^	0.36
Niemeyer et al 2015 [20]	Bovine	A	GP	60–180 W for 2, 4, and 6 min	Plots with diameters	Plots with volumes	–
Cavagnaro et al 2015 [21]	Bovine 32 ablations	B	GP	5 W 10 min; 10 W 10 min; 15 W 10 min; 20 W 10 min; 40 W 10 min	1.40 ± 0.09 × 1.18 ± 0.08 2.18 ± 0.13 × 1.84 ± 0.18 2.51 ± 0.14 × 2.20 ± 0.10 3.17 ± 0.29 × 2.73 ± 0.46 4.33 ± 0.18 × 3.63 ± 0.08	1.02^a^ 3.86^a^ 6.36^a^ 12.37^a^ 25.60^a^	0.71 0.71 0.77 0.74 0.70
Cavagnaro et al 2015 [22]	Bovine	B	GP	60 W 10 min	5.34 ± 1.7 × 4.29 ± 2.0	51.46^a^	0.65
Paul et al 2015 [23]	3 porcine	B	CT	100 W 4.5 min	4.1 ± 0.2 × 5.6 ± 0.2	49.29^a^	0.54
Kim et al 2015 [24]	Bovine	D	GP	Single 24 W 675 s; multi 14-24 W 401 s; single 28 W 339 s; multi 16-28 W 306 s	1.72 × 1.84; 1.56 × 1.69 2.21 × 2.38 1.92 × 2.14	4.63 ± 0.5; 3.75 ± 0.8 15.33 ± 3.4 10.98 ± 2.5	0.87 0.85 0.86 0.80
Pillai et al 2015 [25]	Perfused bovine	D	GP	25-28 W; temperature 110°; 17 min	4.4 ± 0.31 × 5.8 ± 0.4 (− heat sink) 3.6 ± 0.4 × 4.8 ± 0.3 (+ heatsink)	232 ± 28 (− heat sink) 181 ± 21 (+ heatsink)	0.58 0.56
Dodd et al 2015 [37]	Blood-perfused bovine	C	GP	140 W 5 and 10 min	5.61 ± 0.20 × 3.2 ± 0.08; 6.51 ± 0.20 × 3.81 ± 0.07	30.22 ± 1.85; 49.30 ± 1.85	0.33 0.4
Ringe et al 2015 [30]	Perfused porcine 108 ablations	E	GP	45 W 10 min	2.1 × 1.5	2.47^a^	0.51
Weiss et al 2015 [33]	16 bovine livers	B	GP	40 W 1 min 2 min 3 min 6 min 10 min	0.76 1.08 1.30 1.79 2.14		–
Harari et al 2016 [8]	Bovine	C	GP	Simultaneous 2 and 3 antennas 50 W 5 min	3.56 ± 0.39 × 4.51 ± 0.63; 3.97 ± 0.29 × 4.97 ± 0.32;	32.1 ± 5.5; 45.8 ± 8.8;	0.62 0.64
Amabile et al 2016 [11]	20 bovine livers 108 ablations	B	GP	3–30 min 20–130 W	1.6 ± 0.1 × 2.4 ± 0.3–7.2 ± 0.4 × 10.1 ± 0.7	3.22–274.15^a^	0.66–0.70

*A* Acculis, *B* Amica, *C* NeuWave 140, *D* AveCure, *E* Evident, *F* Emprint, *G* MicroThermX, *CT* computed tomography, *MRI* magnetic resonance imaging, *GP* gross pathology

^a^Estimated volume

**Table 3:** Included in vivo studies for qualitative analysis

Author + year	Subjects	MWA system	Ablation zone measured by	Ablation protocol (energy and time)	Ablation diameter (cm)	Ablation volume (mL)	Sphericity index
Hines-Peralta et al 2006 [9]	porcine 14 pigs, 45 ablations	A	GP	50–150 W 4–20 min	2.8 × 4.1–5.8 × 5.5	16.8–110.6^a^	0.35–0.90
Awad et al 2007 [14]	3 porcine 9 ablations	A	GP	100 W 2–8 min	3.7 × 4.5–5.3 × 6.4	33.5 ± 17.3–92.0 ± 6.5	0.60–0.68
Garrean et al 2009 [15]	4 porcine 16 ablations	A	GP	70–100 W 4 min	3.0–6.54	–	–
Meloni et al 2011 [26]	4 porcine 16 ablations	B	GP	40–60 W 15 min	3.3 ± 0.6 × 2.9 ± 0.5–4.2 ± 1.1 × 3.1 ± 1.1	14.5–21.1^a^	0.54–0.77
Lubner et al 2012 [10]	Porcine 48 ablations	C	GP	140 W 2–10 min	2.0 ± 0.2 × 3.2 ± 1.2–3.4 ± 0.6 × 4.1 ± 0.9		0.39–0.69
Correa-Gallego et al 2014 [18]	Porcine 6 ablations	E	GP	45 W 10 min	7.05 cm^2^	–	–
Gockner et al 2015 [19]	3 sheep 9 ablations	A	CT	80 W 2 min	4.15 ± 0.46 × 2.37 ± 0.37	16.5 ± 5.1	0.33
Bedoya et al 2014 [27]	6 porcine	C	GP	5 delivery methods of 30 kJ of energy	2.3 ± 0.7 × 1.4 ± 0.5; 3.5 ± 0.6 × 2.1 ± 0.4; 3.8 ± 0.9 × 2.4 ± 0.7; 4.6 ± 0.6 × 3.0 ± 0.4; 5.2 ± 0.8 × 3.3 ± 0.9	23.6 ± 26.5; 67.6 ± 34.5; 105.4 ± 78.3; 176.7 ± 45.9; 265.7 ± 208.1	0.37 0.36 0.40 0.43 0.40
Moreland et al 2015 [28]	5 porcine 28 ablations	C	GP	65 W 5 min	3.3 ± 0.9	–	–
Harari et al 2016 [8]	Porcine	C	GP	2 and 3 antennas 65 W 5 min	3.33 ± 0.80 × 3.72 ± 0.93; 4.02 ± 0.51 × 4.68 ± 0.44	21.3 ± 13; 47.8 ± 13	0.80 0.74
Amabile et al 2016 [11]	12 porcine 28 ablations	B	GP	5–10 min 60, 80, 100 W	2.5 ± 0.2 × 4.3 ± 0.3–4.9 ± 0.1 × 8.5 ± 0.4	14.07–106.86^a^	0.56–0.66
Wu et al 2016 [34]	4 porcine 15 ablations	C	GP	5 min 100 W	No contrast 23.9 ± 1.2 contrast 22.3 ± 1.8 non-perfused 39.3 ± 1.7		–

*A* Acculis, *B* Amica, *C* NeuWave 140, *E* Evident, *CT* computed tomography, *GP* gross pathology

^a^Estimated volume

**Table 4:** Included clinical studies for qualitative analysis

Author + year	Subjects	MWA system	Ablation zone measured by	Ablation protocol (energy and time)	Ablation diameter (cm)	Ablation volume (mL)	Sphericity index
Ratanaprasatporn et al 2013 [40]	10 ablations and resections (3 HCC; 7 metastatic)	D	GP	10–32 W; 110°–120° 10 min	4.1	8.7	–
Di Vece et al 2014 [42]	20 patients with primary (9) and secondary (11) liver tumors	B	US	60–70 W 10 min	4.85 ± 0.67 × 3.85 ± 0.46	–	0.63
Winokur et al 2014 [16]	36 ablations with Amica (20) and NeuWave (16)	BC	CT	B: 43.8 ± 27.4 kJ C: 21.4 ± 12.6 kJ	B: 5.1 ± 1.5 × 3.0 ± 0.9 C: 3.9 ± 0.7 × 2.7 ± 0.5	B: 33.0 ± 18.9 C: 15.5 ± 6.7	B: 0.49 C: 0.39
Berber et al 2015 [29]	5 patients; 9 malignant tumors	F	CT	Patient specific	Details per ablation	–	–
Berber et al 2016 [31]	18 patients; 54 malignant liver tumors	F	CT	Patient specific	Ablation zone and time of 100 W	–	0.9
Zaidi et al 2016 [32]	53 laparoscopic ablations	F	CT	Patient specific	Ablation time/size plot for 100 W	–	0.9
Amabile et al 2016 [11]	46 patients; 32 HCC; 19 metastasis	B	CT	5 min 60 W 10 min 60 W	HCC 3.3 ± 0.5 × 4.8 ± 0.7 metastasis 4.0 ± 0.9 × 5.5 ± 1.7 HCC 3.7 ± 0.3 × 5.2 ± 0.6 metastasis 4.1 ± 0.6 × 6.5 ± 0.9		HCC 0.64–0.74 metastasis 0.70–0.71
Shyn et al 2016 [36]	52 patients 93 ablations	B	MRI/CT	Patient specific	Correlation with energy		

*B* Amica, *C* NeuWave 140, *D* AveCure, Evident, *F* Emprint, *CT* computed tomography, *MRI* magnetic resonance imaging, *US* ultrasound, GP gross pathology, *HCC* hepatocellular carcinoma

### Ex vivo animal studies

In total, 18 studies published ex vivo animal results, three in porcine liver [23, 30, 39] and 15 in bovine liver (Table 1) [8–12, 17, 20, 22, 24, 25, 33, 35, 37, 38, 41]. Four studies were performed in perfused liver [25, 30, 37, 38]. Dodd et al used 15 blood-perfused (37 °C) bovine livers for 60 MW ablations with system G (MicroThermX) [37]. Ablation zone volumes, measured by gross pathology, were unaffected by changes in portal venous blood flow (range of 60–100 mL/min per 100 g tissue). These authors also repeated the blood-perfused study with 60 ablations in ten livers with system C (NeuWave) [38]. Again, a change in blood flow rate did not affect the size and shape of the ablation zone, as evaluated by pathology. Pillai et al perfused ex vivo bovine livers with 37 °C Ringer solution and compared volumes and diameters of ablation zones in relation to the distance to the major hepatic vein in three ablation experiments [25]. For system D (AveCure), ablation zones within 8 mm to the major hepatic veins were 22% smaller than ablation zones more than 30 mm away from major hepatic veins [25]. Ringe et al used perfused glass tubes in porcine liver to simulate the hepatic veins [30]. They analyzed 108 ablation zones generated by system E (Evident) and found that ablation zones were influenced by flow rate (0, 700, and 1400 mL/min) at a maximum distance of 10 mm to the glass tube. Hoffmann et al compared the four systems A, B, D, and E (Acculis, Amica, AveCure, and Evident) in bovine liver [12]. They found that system C (NeuWave, 3 antennas) created the largest and most spherical zones. Most systems note in their manual that ablation algorithms are based on “internal *ex-vivo* experiments.” However, no peer-reviewed publications were found for ex vivo testing for system F (Emprint).

### In vivo animal studies

In 12 animal studies, the effects of microwave ablation in in vivo liver parenchyma were analyzed [8–11, 14, 15, 18, 19, 26–28, 34]. Gockner et al compared MWA using system A (Acculis) with and without transarterial embolization before ablation in sheep [19]. Extent and shape of the ablation zones were determined by CT. Ablation zone diameters increased by 22.8% by using transarterial embolization before ablation (*p* < 0.01). Hines-Peralta et al compared ex vivo bovine and in vivo porcine MWA, using system A (Acculis) [9]. Unexpectedly, 8-mm larger (57 mm vs. 49 mm) diameter of ablation zones (*p* < 0.01) were obtained in in vivo (57 ± 2 mm) experiments, compared to ex vivo (49 ± 2 mm). However, for ablation times longer than 8 min, ex vivo diameters still increased while in vivo diameters remained constant. Also, Lubner et al compared ex vivo bovine and in vivo porcine MWA [10]. Ablation zones were similar during the first 2 min, but in vivo ablations did not continue to grow as much as ex vivo [10]. No in vivo studies were performed with system D (AveCure) and system F (Emprint).

### Clinical studies

Nine studies reported clinical results of the ablation zone [11, 16, 18, 29, 31, 32, 36, 40, 42]. Ratanaprasatporn et al performed a prospective study in ten patients with liver tumors, treated with system D (AveCure) [40]. After liver resection, the ablation zone volumes were measured on gross pathology. Six of the ten treatments showed ablation with complete necrosis on pathological examination [40]. Di Vece et al reported a mean long-axis diameter of 4.85 cm in 20 patients treated with system B (Amica) [42]. Winokur et al analyzed the ablation properties of system B (Amica, 25 ablations in 20 patients) and system C (NeuWave, 11 ablations in 8 patients) in order to analyze if the manufacturer published reference values are useful [16]. The study indicated that in vivo (clinical) ablation zone volumes are significantly smaller than stated by reference values from the manufacturers (0.69 cm^3^ vs. 1.29 cm^3^; *p* = 0.003) [16]. Berber et al compared predicted ablation diameters (system F, Emprint) with the ablation zone at the 2-week post-ablation CT scan of nine patients [29]. The maximum diameter of the ablation zone was 1.12 ± 0.11 times larger than predicted. No residual tumors were seen at the 2-week scan. In another study, Berber reported a scatterplot showing the CT diameter of ablation zones obtained after 100 W of power during laparoscopic MW ablation with system F (Emprint) in 15 patients [31]. In a study of 149 laparoscopic ablations, Zaidi et al reported system F to be satisfactory in achieving the predicted ablation sizes [32]. Amabile et al compared ablation zone dimensions in liver tumors in patients with both in vivo (porcine) and ex vivo (bovine) experiments (only liver parenchyma). They concluded that the ex vivo animal data reliably predicted the dimensions in (in vivo) human liver tumors. Shyn et al compared ablation zone diameters and volumes with applied and net energy (after correcting for reflectivity), and with manufacturer chart predictions [36]. Applied energy (*r* = 0.52) and net energy (*r* = 0.53) did not correlate better than manufacturer chart prediction (*r* = 0.60). Also, no differences were seen between cirrhotic and non-cirrhotic livers. None of the clinical studies were performed with system A (Acculis), E (Evident), and G (MicroThermX).

### Comparison of ablation volume–applied energy ratio

**Fig. 2:**
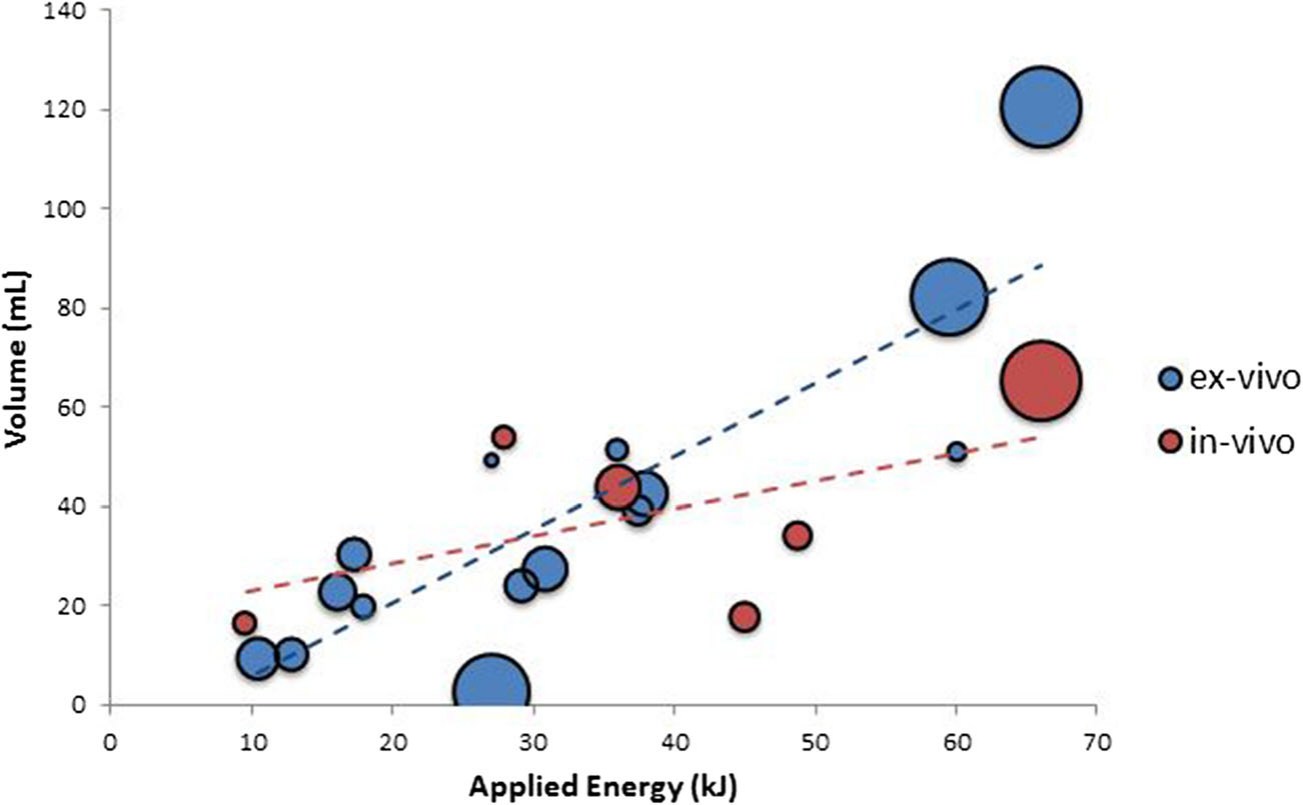
Bubble chart of the ratio of ablation zone volume (mL) to applied energy (kJ) R(AZ:E) for 22 subgroups in animal studies of all devices with adequate volume/energy representation. The sizes of the bubbles are determined by the sample size of the subgroup

In total, 14 animal studies reported data on ablation zone volume and applied energy and could be used to compare R(AZ:E) [8, 9, 11, 12, 14, 17, 19, 22–24, 26, 30, 35, 39]. These studies were categorized in 22 subgroups based on device and tissue (ex vivo and in vivo). A significant correlation between volume and energy was found for the ex vivo experiments (*r* = 0.85, *p* < 0.001) in contrast to the in vivo experiments (*r* = 0.54, *p* = 0.27) (Fig. 2). Results of SI calculation are shown in Tables 2, 3, and 4.

## Discussion

In this review on studies performed with seven FDA-approved MWA devices, we found that only a minority (9 out of 43 studies) was based on clinical studies. On top of that, a major limitation in these clinical studies is the lack of data on the ratio ablation zone volume: applied energy R(AZ:E). In only 14 preclinical studies, the R(AZ:E) was calculated. To our knowledge, this is the first study which compares the ratio ablation zone volume and applied energy of FDA-approved MWA devices between 2005 and 2017. MWA systems are categorized as class II medical devices by the FDA. This classification requires that a new device must be proven to be substantially equivalent to a device that was legally marketed (predicate device) prior to May 28, 1976. Since microwave technology has been around for decades, ex vivo bench testing is sufficient for the regulatory approvals and no additional submission of clinical data is required [7]. Also, reporting results on ablation zone volume and applied energy in clinical setting are limited. This might be the reason for the lack of clinical data of MWA systems.

An analysis of the studies in our review suggests a number of possible explanations for the discrepancy between manufacturers’ provided ablation algorithms and the actual clinical ablation zone sizes. This discrepancy was demonstrated by Winokur et al who indicated that in vivo (clinical) ablation zone volumes are significantly smaller than stated by reference values from the manufacturers of the Amica and NeuWave system [16]. Depending on the type of study (ex vivo vs. in vivo), ablation time was found to be a determinant for ablation zone diameter. For ablation time longer than 8 min, ex vivo diameter still increased while in vivo diameters remained constant, suggesting plateau formation which probably is caused by the antagonizing effect of perfusion [9, 16].

Obviously, preclinical studies using animal livers are only performed in normal liver parenchyma with absence of tumor tissue (tumor characteristics) and underlying liver disease (liver characteristics). Deshazer et al simulated these properties in a two-compartmental computer model and showed that ablation zone volume could increase with 36% in patients with cirrhotic liver as compared to healthy liver tissue [6]. Tumor tissue revealed a 20% higher thermal conductivity (the property of tissue to conduct heat) than healthy liver tissue. Hyperperfused and hypoperfused tumors within normal liver parenchyma showed minimal variation in ablation zone volume. Furthermore, steatotic parenchyma had 50% lower thermal conductivity than healthy liver tissue [6]. Hepatic steatosis is of importance in patients with CRLM treated with neo-adjuvant chemotherapy and should be taken into account when planning MWA treatment for these patients [43]. Perfusion in ex vivo experiments also determines to a great extent the effect of heat distribution by convection in MWA; convection of heat is mainly influenced by vascularization of the tumor and adjacent large blood vessels which can cause heat sink. In most ex vivo studies, livers were not perfused and thus not subject to heat sink effects. Perfusion of cirrhotic liver tissue is reported to be 36% lower than healthy liver tissue [44]. This is of importance because approximately 90% of the patients with HCC suffer from cirrhosis [45]. Additionally, the majority of HCCs have a predominant arterial perfusion, which makes prediction of the obtained ablation zone in relation to perfusion phenomena even more imprecise. Compared to perfusion in liver parenchyma, HCC tumor tissue has a significantly higher arterial perfusion and lower portal venous hepatic blood flow [46]. Therefore, the results of Amabile et al are difficult to interpret, because they found a better correlation between ablation zone dimensions in liver tumors (HCC) in patients and non-perfused (ex vivo) bovine liver than in perfused (in vivo) porcine liver. A possible explanation for this seemingly contradictory finding is that less than 20% of the ablation zone volume encompasses tumor and more than 80% liver parenchyma [47]. This suggests that liver parenchyma might be of more importance for the ablation zone volume than the tumor tissue. Clinical studies were limited in number and additionally in general no distinction in tumor type (primary or secondary) is made. None of the studies in this review reported the underlying liver diseases, like hepatic steatosis, fibrosis, or cirrhosis. In a retrospective study, MWA volumes of HCC in cirrhotic liver and CRLM in healthy liver were compared. R(AZ:E) for HCC with system A (Acculis) is twofold higher for HCC (R(AZ:E) = 0.61) compared to CRLM (R(AZ:E) = 0.35). Also, ablation treatment for HCC with system B (Amica) resulted in a 50% increase of ablation zone volume, compared to CRLM [47]. For treatment of HCC up to 4 cm, no significant differences were found in local tumor progression between radiofrequency ablation (RFA) and MWA [48].

During liver ablation, the power of heating (Watt) is an important factor. When using low power, relatively more heat will be dispersed into the surrounding tissue, and temperature of the ablation zone will be low. In contrast, higher power for short time will lead to high temperatures around the antenna and contraction of the target tissue. Bedoya et al compared different ways to deliver 30 kJ of energy (25 W 20 min, 50 W 10 min, and 100 W 5 min) in in vivo porcine livers [27]. Significantly larger ablation zone volumes were achieved with high power ablations (23.6 ± 26.5 mL; 105.4 ± 78.3 mL; 265.7 ± 208.1, respectively; *p* < 0.03). Interestingly, they also investigated the effect of pulsed energy delivery (25 kJ) to limit the effects of heat sink and to provide larger ablation zone volumes than continuous energy delivery (67.4 ± 34.5 cm3 vs. 23.6 ± 26.5 cm3, *p* = 0.43).

Another important factor is the reflection of energy by the antenna cable. The majority of the microwave systems report the output power of the generator as the applied energy at the antenna. However, 15–30% of the output energy per meter length will be lost by the antenna cable [49]. So, for comparison of ablation devices by R(AZ:E), the most relevant parameter is the amount of energy deposited into the liver tissue, which is known as the net energy [5, 49, 50]. Only systems B (Amica) and D (AveCure) display the reflection of energy on the generator. A comparable disagreement is found for the various antenna designs: if an antenna is built to have stronger fields in certain areas (Watts/area), a different amount of tissue reaching 60 °C might be expected. So, differences in R(AZ:E) between devices might be due to antenna design and cable length [49]. Despite the limitation of using R(AZ:E), it is the only parameter to compare the currently available data quantitatively.

Most liver tumors treated by MWA are spherical which requires also a spherical ablation zone to achieve a sufficient ablation margin. However, most ablation systems create ellipsoidal ablation zones with poor sphericity values. Reflection of energy by the antenna shaft results in heating of the shaft which contributes to bigger LAD. This may also increase the risk of thermal damage of adjacent liver tissue. Data about the sphericity of ablation zones in patients are scarce. In this study, we calculated the SI for all studies in which the LAD and SAD were reported. However, data are heterogeneous and depending on power and time of the ablation. A recently published study compared system F (Emprint) with systems B (Amica) and E (Evident) [51]. Significantly more spherical ablation zones in patients were achieved with system F than with systems B and E. Complete ablation was possible with a single antenna placement regardless of the angle approach because of the almost spherical ablation zone.

There might be several strategies to decrease ASR after microwave ablation. First of all, more in vivo and clinical studies with MWA should be conducted with cirrhotic and other diseased parenchyma, like steatotic liver, to confirm computer model studies. Secondly, presentation of data in studies should be reported adequately by means of applied energy (without reflection), LAD, SAD, SI, and volume of each ablation zone (Table 5). Additionally, for clinical studies, the status of the underlying liver parenchyma should be reported.

**Table 5:** Guidelines for reporting on future studies describing ablation experiments

Variable	Value
Type of experiments	Ex vivo (perfused, non-perfused), in vivo, clinical
Subjects	animal (porcine, bovine), human
Type of liver parenchyma	normal, cirrhosis, fibrosis, steatotic
Device	
Applied energy (kJ)	Energy = ablation time (seconds) × power (Watt) / 1000
Ablation diameters (cm)	Long-axis diameter (LAD) and short-axis diameter (SAD)
Ablation zone volume (mL)	$V=\frac{4}{3}\pi \left( LAD/2\right){\left( SAD/2\right)}^2$
Sphericity index	*SI* = *SAD*^2^/*LAD*^2^

*LAD* long-axis diameter, *SAD* short-axis diameter

There are some limitations to this study. Ablation outcomes were compared by R(AZ:E). Ablation zone volume as reported in the selected papers was recorded. If only one or two diameters were reported, the ablation zone volume was estimated assuming ellipsoid or spherical morphology. Also, the diameters or volumes of the ablation zones were assessed by different measurement modalities (gross pathology, CT, MRI, or ultrasound) which induce bias. Finally, a systematic review formally requires a control group, but it is clear that this is not available for the current review.

In conclusion, manufacturers’ algorithms on microwave ablation zone sizes are based on preclinical animal experiments with normal liver parenchyma. Clinical data reporting on ablation zone volume in relation to applied energy and sphericity index during MWA are scarce which requires more adequate reporting on MWA data.

## Abbreviations

ASR: Ablation site recurrences
CRLM: Colorectal liver metastases
FDA: US Food and Drug Administration
HCC: Hepatocellular carcinoma
LAD: Long-axis diameter
MWA: Microwave ablation
R(AZ:E): Ratio of ablation zone volume to applied energy
SAD: Short-axis diameters
SI: Sphericity index
